# A48 PARENT-REPORTED HEALTH RELATED QUALITY OF LIFE CARING FOR CHILDREN WITH PEDIATRIC FEEDING DISORDERS IN ALBERTA

**DOI:** 10.1093/jcag/gwad061.048

**Published:** 2024-02-14

**Authors:** A Lang, A Chui, J Cohen, V Steinke, M Moland, J Turner

**Affiliations:** . Pediatrics, University of Alberta, Edmonton, AB, Canada; . Alberta Health Services, Edmonton, AB, Canada; . Alberta Health Services, Edmonton, AB, Canada; . Alberta Health Services, Edmonton, AB, Canada; . Alberta Health Services, Edmonton, AB, Canada; . Pediatrics, University of Alberta, Edmonton, AB, Canada

## Abstract

**Background:**

Children with pediatric feeding disorders (PFD), including those requiring tube feeding, need significant care. Caregiver burden includes time, cost, as well as worry over patient morbidity and mortality (1). This project used a validated health-related quality of life (HRQoL) tool for caregivers of children with PFD: the Feeding/Swallowing Impact Survey (FS-IS). The FS-IS has three subscales related to daily activities, worry, and feeding concerns, scored 1-5 with 5 being the most adverse. Prior studies have shown both the total sub-scale scores and the total score correlate with generic quality of life instruments for pediatric caregivers (2). Data was obtained from a provincial quality improvement initiative, the Alberta Provincial Pediatric Eating and Swallowing (PEAS) Project.

**Aims:**

1. To determine the HRQoL reported by caregivers of children with PFD in Alberta. 2. To determine if tube-feeding is associated with worse reported HRQoL.

**Methods:**

Caregivers receiving feeding and swallowing services in Alberta were offered the opportunity to complete the FS-IS online or by phone at baseline and 12 months later. All demographic data was self-reported.

**Results:**

In total 52 caregivers completed the surveys once and 16 at follow-up. Average baseline FSIS was 3.1 (range 1.2-4.7) and at follow up was 3.0 (range 1.5-4.2). There was no difference between the scores of those completing at both time points (p=0.78). At all time points, only 15% of the cohorts reported scores ≤2. As shown in Table 1, children who were tube-fed were older with a longer duration of PFD symptoms, however, HRQoL scores were not different to children not ever tube-fed.

**Conclusions:**

Caregivers of children with PFD face challenges, which do not abate over time. Surprisingly, in our cohort, this is independent of the need for tube feeding and of the duration of PFD symptoms, although we acknowledge the small sample size of those who were never tube-fed. Longitudinal follow-up studies and strategies to improve quality of life are needed.

* FS-IS Global Score (1=never to 5=almost always)

**Funding Agencies:**

Alberta Health Services Maternal Newborn Child & Youth Strategic Clinical Network

**Table:** 

	Tube Fed Ever (n=46)	Never Tube Fed (n=6)	p-value
Female (%)	39	33	0.75
Age (months)	56	23	0.001
Duration of PFD symptoms (months)	52	20	0.001
Initial FS-IS score	2.9	3.2	0.43

